# Differential Regulation of NAPDH Oxidases in Salt-Tolerant *Eutrema salsugineum* and Salt-Sensitive *Arabidopsis thaliana*

**DOI:** 10.3390/ijms221910341

**Published:** 2021-09-25

**Authors:** Maria Pilarska, Dorothea Bartels, Ewa Niewiadomska

**Affiliations:** 1 The Franciszek Górski Institute of Plant Physiology, Polish Academy of Sciences, Niezapominajek 21, 30-239 Kraków, Poland; e.niewiadomska@ifr-pan.edu.pl; 2 Institute of Molecular Physiology and Biotechnology of Plants (IMBIO), University of Bonn, Kirschallee 1, 53115 Bonn, Germany; dbartels@uni-bonn.de

**Keywords:** halophyte species, NADPH oxidases, NOX, respiratory burst oxidase homolog *RBOH* gene expression, saline adaptations

## Abstract

Reactive oxygen species (ROS) signalling is crucial in modulating stress responses in plants, and NADPH oxidases (NOXs) are an important component of signal transduction under salt stress. The goal of this research was to investigate whether the regulation of NOX-dependent signalling during mild and severe salinity differs between the halophyte *Eutrema salsugineum* and the glycophyte *Arabidopsis thaliana*. Gene expression analyses showed that salt-induced expression patterns of two NOX genes, *RBOHD* and *RBOHF*, varied between the halophyte and the glycophyte. Five days of salinity stimulated the expression of both genes in *E. salsugineum* leaves, while their expression in *A. thaliana* decreased. This was not accompanied by changes in the total NOX activity in *E. salsugineum*, while the activity in *A. thaliana* was reduced. The expression of the *RBOHD* and *RBOHF* genes in *E. salsugineum* leaves was induced by abscisic acid (ABA) and ethephon spraying. The in silico analyses of promoter sequences of *RBOHD* and *RBOHF* revealed multiple *cis*-acting elements related to hormone responses, and their distribution varied between *E. salsugineum* and *A. thaliana*. Our results indicate that, in the halophyte *E. salsugineum*, the maintenance of the basal activity of NOXs in leaves plays a role during acclimation responses to salt stress. The different expression patterns of the *RBOHD* and *RBOHF* genes under salinity in *E. salsugineum* and *A. thaliana* point to a modified regulation of these genes in the halophyte, possibly through ABA- and/or ethylene-dependent pathways.

## 1. Introduction

Soil salinity is one of the major environmental factors that restrict crop productivity and the functioning of plants in natural ecosystems [1,2]. A common reaction of plants to salt-triggered osmotic stress and ionic imbalance is to increase the levels of reactive oxygen species (ROS) [3,4]. These reactive species include superoxide (O_2_^•−^), hydroxyl radical (OH^•^), hydrogen peroxide (H_2_O_2_), and singlet oxygen (^1^O_2_) and are generated constantly as products of normal cell metabolism. For a long time, ROS were thought to have primarily negative effects on the cells, such as lipid peroxidation, protein denaturation, and DNA damage [5]. However, more recently, ROS have been perceived as universal signalling metabolites regulating plant growth, development, and defence against biotic and abiotic stresses [6,7,8]. ROS are generated in several cellular compartments, such as chloroplasts, mitochondria, and peroxisomes. In the plasma membrane, ROS are synthesised by NADPH oxidases (NOXs), also termed respiratory burst oxidase homologs (RBOHs) [9]. These enzymes with homology to the NADPH oxidase from mammalian phagocytes can use NADPH as an electron donor to reduce O_2_ molecules and to generate O_2_^•−^ in the apoplast. The resulting O_2_^•−^ can be converted into H_2_O_2_ spontaneously or enzymatically by superoxide dismutase (SOD) [9,10]. NADPH oxidases in plants constitute a multigene family and the *Arabidopsis* *thaliana* genome contains 10 genes, *RBOHA-J*, which exhibit a different pattern of expression during ontogenesis and in response to stress stimuli [11,12]. The results of extensive studies confirmed that ROS produced by NOXs regulate many physiological processes, such as seed germination [13], stomatal opening [14], root growth [15], and pollen tube elongation [16]. RBOH-dependent ROS also appear to play an important role in signalling networks enabling acclimation to various stresses, including heat [17], wounding [18], drought [19], and salinity [20,21]. 

The regulatory mechanisms of NOXs are still under extensive research and their activity is controlled by different factors, such as calcium and protein kinases [22,23]. A link has been established between phytohormones and the regulation of NADPH oxidases. Abscisic acid (ABA) induced the expression of two *RBOH* genes in *A. thaliana* guard cells [14], and the overexpression of the ABA biosynthesis gene in tobacco resulted in salt-tolerance associated with NOX-dependent ROS production [24]. 

The two pleiotropic NOX genes in *A. thaliana*, *RBOHD* and *RBOHF*, are the main NADPH oxidases associated with acclimation to salinity [20]. It has been shown that an early response to salt stress is a calcium wave, which depends on the ROS produced by RBOHD and which then propagates the systemic response to salt [25]. The analyses of *A. thaliana* mutants enables linking the ROS produced by RBOHD and RBOHF with the regulation of Na^+^/K^+^ homeostasis under salinity, which relates to salt resistance [20,26]. The RBOHD- and RBOHF-related ROS also enhanced the accumulation of proline, an osmolyte associated with salinity tolerance [27]. 

Salt stress is harmful to most plants except for some species, termed halophytes, which can grow and reproduce in high salinity environments [28]. Halophytes activate protective mechanisms to prevent high Na^+^ accumulation in the cytosol and to maintain photosynthesis [29,30,31]. The salt-tolerance of halophytes seems to be linked with their ability to control the redox balance [3,32,33]. The comparison of two close relatives, the glycophyte *Arabidopsis thaliana* and the halophyte *Eutrema salsugineum*, showed that the latter was capable of enhancing the production of H_2_O_2_ under control and stress conditions [34,35]. This suggests a different regulation of ROS formation in these two species. The effect of H_2_O_2_ signalling depends not only on its type but also on the site of its origin in the cell, as shown for *A. thaliana* [36]; therefore, precise control of ROS formation may contribute to the outcome of the plant stress responses.

The goal of this research was to investigate whether the regulation of NOX-dependent signalling under salinity differs between the glycophyte *A. thaliana* and the halophyte *E. salsugineum*. We aimed to characterise the expression patterns of the *RBOHD* and *RBOHF* genes and total NADPH oxidase activity in *A. thaliana* and *E. salsugineum* during five days of salinity treatment. To gain insight into possible hormonal regulation mechanisms of *RBOHD* and *RBOHF*, we monitored the gene expression in response to ABA and ethephon and performed an in silico search for *cis*-acting promoter elements.

## 2. Results

The *E. salsugineum* genome was searched using the available genome sequences of *RBOHD* and *RBOHF* in *A. thaliana*, and one homolog of each gene was found. The conserved domains characteristic of the RBOH family [37] were searched after aligning the amino acid sequences of RBOHD and RBOHF in *E. salsugineum* with the homologs in *A. thaliana*. The C-terminal region included the FAD-binding domain and ferric reductase NAD binding domain (Supplementary Figure S1). The N-terminal regions of the predicted EsRBOHD and EsRBOHF proteins contained the respiratory burst NADPH oxidase domain and putative Ca^2+^-binding EF hands.

**Figure 1:**
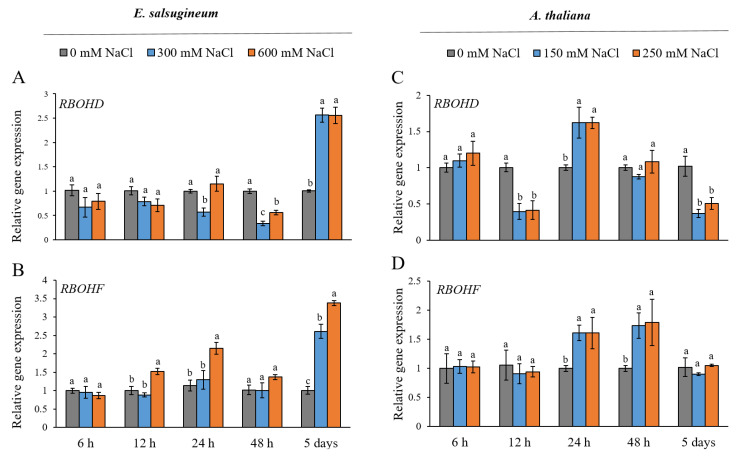
Gene expression patterns of *RBOHD* (**A**,**C**) and *RBOHF* (**B**,**D**) in leaves of *E. salsugineum* and *A. thaliana* under NaCl stress conditions. *E. salsugineum* plants were exposed to 0, 300, or 600 mM NaCl and *A. thaliana* plants were exposed to 0, 150, or 250 mM NaCl for 6, 12, 24, or 48 h or 5 days. Data represent mean ± SE (*n* = 3). Different letters illustrate significant differences at *p* ≤ 0.05.

To establish whether the *RBOHD* and *RBOHF* genes in leaves of *E. salsugineum* respond to short (between 6 and 48 h) and prolonged (5 days) salinity conditions, the expression patterns were examined under moderate and severe NaCl stress and compared with the response of homologous genes in *A. thaliana*. The first significant changes in the expression of *RBOHD* in *E. salsugineum* were detected after 24 h of salt-stress. At this time, mild salinity suppressed the accumulation of the *EsRBOHD* transcripts (Figure 1A). Mild and severe salt treatment downregulated the *RBOHD* in this halophyte after 2 days, and the suppression was stronger under 300 mM NaCl. After 5 days of salinity conditions, the expression of the *RBOHD* gene increased (over 2-fold) regardless of the salt concentration used. Only under severe salt stress, 600 mM NaCl, was *RBOHF* in *E. salsugineum* upregulated at 12 h (less than 2-fold) and at 24 h (less than 2.5-fold) (Figure 1B). The *EsRBOHF* transcript also accumulated after 5 days of salinity but at a slightly higher level in response to 600 mM NaCl conditions (over 3.5-fold increase) than to 300 mM NaCl (over 2.5-fold increase). 

In *A. thaliana*, a 12-h exposure to moderate and severe salinity suppressed the expression of the *AtRBOHD* gene, but at 24 h, a slight upregulation was observed (less than 2-fold) (Figure 1C). After 5 days of mild and severe salt stress, the level of the *RBOHD* transcripts was significantly lower in relation to respective controls. Under both salinity treatments, the upregulation of *AtRBOHF* (less than 2-fold) was observed after 24 and 48 h (Figure 1D). The level of the *AtRBOHF* transcripts was not different from control samples after 5 days of salinity conditions. 

**Figure 2:**
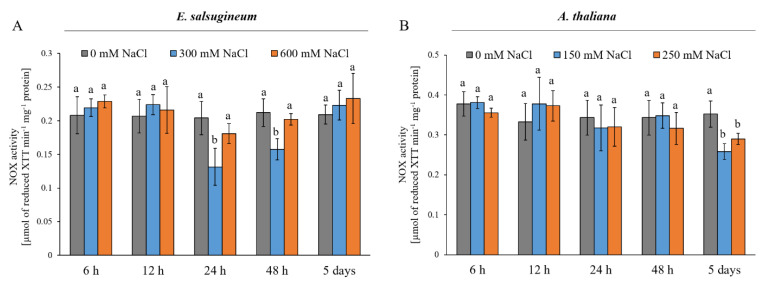
Total activity of NOX in leaves of *E. salsugineum* (**A**) and *A. thaliana* (**B**) plants under NaCl stress conditions. *E. salsugineum* plants were exposed to 0, 300, or 600 mM NaCl and *A. thaliana* were exposed to 0, 150, or 250 mM NaCl for 6, 12, 24, or 48 h or 5 days. Data represent mean ± SD (*n* = 3). Different letters illustrate significant differences at *p* ≤ 0.05.

Next, we aimed to verify whether changes in the transcript levels of the *RBOHD* and *RBOHF* genes in *E. salsugineum* and *A. thaliana* leaves under salinity translated to the activity of the enzymes; therefore, the total activity of NOXs in the leaf microsomal fraction was analysed. In *E. salsugineum*, the activity was decreased at 24 and 48 h of 300 mM NaCl conditions by 35.6 % and 11.3 %, respectively (Figure 2A). The activity of NADPH oxidase in the halophyte was not different from the control after 5 days of salinity, whereas in *A. thaliana*, changes in the activity of NOX were detected after 5 days of mild and severe salinity. The activity decreased by 27 % in plants treated with 150 mM NaCl and by 17.6 % in plants treated with 250 mM NaCl (Figure 2B). 

**Figure 3:**
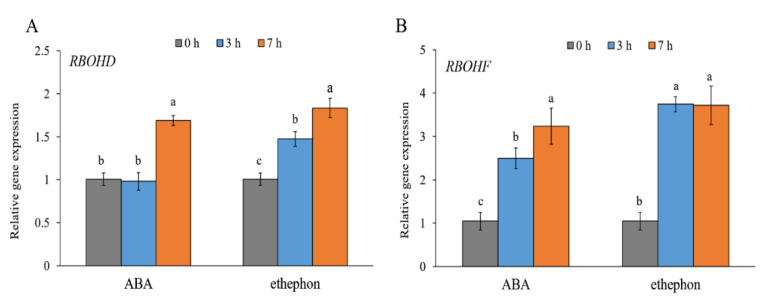
Gene expression patterns of *RBOHD* (**A**) and *RBOHF* (**B**) *in E. salsugineum* leaves sprayed with ABA or ethephon. Data represent mean ± SE (*n* = 3). Different letters illustrate significant differences at *p* ≤ 0.05.

To evaluate the response of the *RBOHD* and *RBOHF* genes in *E. salsugineum* to exogenously applied stress hormones, the leaves were sprayed with ABA or ethephon, which quickly converts to ethylene [38]. As shown in Figure 3A, *RBOHD* was slightly induced by ABA after 7 h (less than 2-fold). The *RBOHD* transcripts were increased (less than 2-fold) at 3 and 7 h after the ethephon treatment. Both ABA and ethephon induced the expression of *RBOHF* by more than 2-fold at 3 h and by over 3-fold at 7 h after spraying of the leaves (Figure 3B). 

To gain an insight into possible transcriptional regulation of the *RBOHD* and *RBOHF* genes in *A. thaliana* and *E. salsugineum*, their promoter sequences were scanned using the PLACE database to predict possible *cis*-acting regulatory elements. Considering that the influence of stress-mediating hormones on the expression of the *RBOH* genes in plants has been reported earlier [14,39], we focused on the presence of *cis*-elements related to hormone signalling in 1500 bp upstream region from the transcription start site. The promoters of *RBOHD* and *RBOHF* were enriched in *cis*-elements responsive to salicylic acid, ethylene, cytokinins, auxins, gibberellins, and ABA (Table 1). However, the distribution of most identical sites varied between *E. salsugineum* and its homologs in *A. thaliana*. In all analysed promoters, multiple motifs involved in responses to ABA and gibberellins were localised. Conversely, only one motif was predicted for ethylene (ERELEE4) and cytokinins (CPBCSPOR) in all the promoters. The ABA responsive *cis*-element, LTRECOREATCOR15, was predicted only in *AtRBOHD* and *AtRBOHF*, while another ABA responsive motif, RYREPEATVFLEB4, was limited to *EsRBOHF*. Additionally, one auxin responsive motif, CATATGGMSAUR, was observed only in *EsRBOHD*. 

## 3. Discussion 

Salt-sensitive and salt-tolerant plant species use generally the same basic mechanisms of adaptation to salinity; therefore, it has been postulated that the differences in salt tolerance are most probably based on specific regulatory mechanisms [40]. As ROS signalling is a crucial component modulating stress responses in plants [3,7,41], it is reasonable to speculate that differences in the regulation of its components, such as NOXs, might influence stress tolerance. To verify this hypothesis, we compared the expression profiles of two NOX genes important in ROS signalling under salt-stress, *RBOHD* and *RBOHF*, in the halophyte *E. salsugineum* and the glycophyte *A. thaliana*. Our results showed that short-time NaCl conditions, up to 48 h, were accompanied by the increased activity of *RBOHF* in both species. Thus, in *E. salsugineum*, similar to *A. thaliana*, *RBOHF* might be involved in the early acclimation of leaves to salinity. However, in our studies, in contrast with *A. thaliana*, the early transcriptional changes of the *RBOHF* gene in *E. salsugineum* were dependent on NaCl concentrations since only severe salinity triggered the gene expression at 12 and 24 h of exposure to salt. A similar observation was made for the expression of aldehyde dehydrogenase genes, where high salt concentrations were required to trigger the defence mechanisms in *E. salsugineum* compared with *A. thaliana* [42]. In *A. thaliana*, the *RBOHD* and *RBOHF* genes were previously assigned to play a role in the primary responses to NaCl, since the stimulation of their expression was observed in seedlings within a few hours of the stress treatment [20,43]. In agreement with this view, we also detected the salinity-induced activation of *AtRBOHD* after 24 h of the stress treatment. Salinity conditions lasting for 5 days stimulated an expression of *RBOH* genes in *E. salsugineum*, which stood in contrast to *A. thaliana*. This suggests that, in the leaves of the halophyte, the *RBOHD* and *RBOHF* genes might be involved in the late acclimation response to ionic and osmotic stress.

We demonstrated that the total activity of NADPH oxidases in *E. salsugineum* was not affected by severe salt stress. Prolonged mild salinity also did not trigger changes in the enzyme activity despite an initial decline. It may be concluded from the activity measurements that the acclimation to salinity was associated with maintaining the basal activity of NOXs in the halophytic *E. salsugineum*, while the activity declined in *A. thaliana*. These observations are in agreement with Srivastava et al. [44] who showed that the total NADPH oxidase activity in the halophyte *Sesuvium portulacastrum* was unaffected, while it decreased significantly in the glycophyte *Brassica juncea*. The discrepancy of the *EsRBOHD* and *EsRBOHF* transcript level and enzyme activities after 5 days of salinity may result from post-transcriptional regulation of the RBOH proteins. Such a phenomenon was reported earlier for NOXs in cucumber [45]. The molecular mechanisms of NOXs activation are complex, which might explain the lack of stimulation in the in vitro activity assays. It is known that these enzymes are subjected to activation depending on Ca^2+^ and phosphatidic acid and are modified by various kinases [22,23,46]. 

The ABA and ethylene signalling pathways have been associated with the acclimation of plants to salinity [47,48]. In our studies, the *EsRBOHD* and *EsRBOHF* genes were induced by ABA and ethephon, which indicates a regulation of NOXs in *E. salsugineum* through the ABA- and ethylene-dependent pathways. In *A. thaliana*, the ABA caused a suppression of the *RBOHD* gene, whereas *RBOHF* was suppressed by ethylene [12,49]. Our observations contrast these results. These differences between species in the regulation of the *RBOHD* and *RBOHF* genes might influence their responses to the salt treatment. Earlier studies documented that the ethylene precursor was increased due to salinity in *E. salsugineum* and *A. thaliana* [35,50]. A significant increase in ABA was detected only in *A. thaliana* leaves as a result of exposure to salinity, while only a slight or no increase in ABA levels was observed in *E. salsugineum* [35,51,52].

The in silico analyses of *cis*-acting elements in promoters of the *RBOHD* and *RBOHF* genes in *E. salsugineum* and *A. thaliana* indicated a possible regulation by stress-related hormones (ABA, ethylene, salicylic acid, and jasmonic acid) and by growth-stimulating hormones (auxins, cytokinins, and gibberellins). It is in agreement with earlier studies, where multiple *cis*-acting elements related to hormonal regulation were predicted in promoters of the *RBOH* genes in *A. thaliana* and rice, including ethylene, ABA, auxins, and salicylic acid [12]. Our studies showed that the number of predicted *cis*-acting elements varied between the analysed homologs, which points to differences in the promoter architecture and possibly promoter activity. 

## 4. Methods

### 4.1. Plant Material, Growth Conditions, and Treatments

The seeds of *Eutrema salsugineum* (earlier *Thellungiella salsuginea*) ecotype Shandong and *Arabidopsis thaliana* ecotype Columbia were obtained from Nottingham Arabidopsis Stock Centre (University of Nottingham, Loughborough, UK). The plants were individually grown in 100 mL pots containing market available soil (pH 5.5–6.5, NaCl < 1.9 g dm^−3^; Verve, Greenyard Horticulture, Pasłęk, Poland) and were irrigated with tap water (the water quality parameters are listed in Supplementary Table S1). Both species were cultivated in a growth chamber at photoperiod 10/14 h, an irradiance of about 120 μmol m^−2^ s^−1^, temperatures of 23/20 °C day/night, and 55–65% relative humidity. Plants with fully developed rosette leaves were used for salt stress and hormone treatment. The salt stress was applied by daily irrigation with 20 ml of the NaCl solution (Sigma-Aldrich, St. Louis, MO, USA). Taking into consideration different salt sensitivities of the species used in this study, the concentration of NaCl was different for *E. salsugineum* and *A. thaliana*. To induce mild stress, 300 mM NaCl was used for the former and 150 mM NaCl was used for the latter species. To induce severe salt stress, 600 mM NaCl was used for *E. salsugineum* and 250 mM NaCl was used for *A. thaliana*. Plants irrigated with 20 ml of water were treated as the control. The leaves were collected at 6, 12, 24, 48 h, and 5 days after the onset of NaCl treatment, used immediately or frozen in liquid nitrogen, and kept at −80 °C for further analysis. For spraying the leaves of *E. salsugineum*, 400 mM ABA (Sigma-Aldrich) [49] or 7 mM ethephon (Sigma-Aldrich) [38] dissolved in ethanol was used. Mock-treated plants were sprayed with 1 % ethanol solution. The leaves were collected 3 and 7 h after the spraying, immediately frozen in liquid nitrogen, and kept at −80 °C for further analysis. 

### 4.2. Database Search and Prediction of Cis-Acting Regulatory Elements

The sequences of the *EsRBOHD* and *EsRBOHF* genes were retrieved from the Phytozome v12.1 database (http://phytozome.jgi.doe.gov/; accessed on 23 February 2018) after the genome of *Eutrema salsugineum* was searched using the *Arabidopsis thaliana RBOHD* (At5g47910) and *RBOHF* (At1g64060) sequences as queries. The Phytozome ID were Thhalv10003619m for *EsRBOHD* and Thhalv100023240m for *EsRBOHF*.

The PLACE online tool [53] was used to predict *cis*-acting regulatory elements in the promoter region (1500 bp upstream region from transcription start site) of *RBOHD* and *RBOHF* in *A. thaliana* and *E. salsugineum*.

### 4.3. Gene Expression Analysis 

Total RNA was extracted from the frozen leaf tissue according to Valenzuela-Avendaño et al. [54]. RNA purity and quantity were determined by Biospec-Nano (SHIMADZU, Kyoto, Japan). The integrity of the RNA samples was assessed on a 2.0 % (*w/v*) agarose gel. RNA samples were treated with DNase I (Thermo Fisher Scientific, Waltham, MA, USA) to remove any traces of DNA. To produce a single-stranded cDNA population, 2 μg of total RNA were reversely transcribed with a RevertAid First Strand cDNA Synthesis Kit (Thermo Fisher Scientific), using the oligo (dT)_18_ primer technique according to the manufacturer’s instructions. Relative quantitative real-time polymerase chain reaction (qRT-PCR) was performed with Maxima SYBR Green Master Mix (Thermo Fisher Scientific) using a CFX96 Touch Real-Time PCR Detection System (Bio-Rad, Hercules, CA, USA). Each qPCR analysis was performed for three samples of each variant and three technical replicates of each sample. The transcript levels were normalised to adenine phosphoribosyl transferase 1 (*APT1*) in the case of *E. salsugineum* and ubiquinol-cytochrome C reductase iron-sulphur subunit (*AT5G*) in the case of *A. thaliana* [21]. The gene sequences were obtained from the Phytozome database as described above. The primers used are listed in Supplementary Table S2. The probability of secondary structure folding in resulting target sequences was predicted with the M-fold webserver [55]. Reaction efficiency was tested by serial dilutions of cDNAs with gene-specific primers and the primer specificities were confirmed with the melting-curve analysis after amplification during the subsequent qPCR analysis. The expression was calculated according to Pfaffl [56], with water-treated plants serving as the calibrator.

### 4.4. Membrane Protein Extraction

Freshly harvested leaves were homogenised in a protein extraction buffer containing 50 mM Tris-HCl, 0.25 M sucrose, 2.5 mM dithiothreitol, and 0.1 mM MgCl_2_ under chilled conditions. The homogenate was filtered through a cheesecloth and centrifuged at 10,000× *g* for 15 min at 4 °C. The microsomal fraction was separated from the supernatant by centrifugation at 80,000× *g* for 45 min at 4 °C, according to the procedure described by Janeczko et al. [57]. The protein content was measured according to the method of Bradford [58] using BSA as a standard. All chemicals used were purchased from Sigma-Aldrich.

### 4.5. NADPH Oxidase (NOX) Activity

The total NOX activity was determined by measuring the reduction of 2,3-*bis*(2-methoxy-4-nitro-5-sulfophenyl)-2H-tetrazolium-5-carboxanilide inner salt (XTT; BioShop, Burlington, ON, Canada) by O_2_^•−^ radicals at 470 nm [59,60]. The reaction mixture contained 50 mM Tris-HCl (Sigma-Aldrich) pH 7.5, 0.5 mM XTT, 0.6 mM NADPH (Sigma-Aldrich), and 5 µg of membrane proteins. The reaction was started by the addition of the NADPH solution. Measurements of absorbance changes in the presence and absence of 50 U SOD (Sigma-Aldrich) were carried out at 470 nm (for XTT ɛ = 2.16 × 10^4^ M^−1^ cm^−1^) using an xMark™ Microplate Absorbance Spectrophotometer (Bio-Rad). Enzyme activity was defined as 1 μmol of XTT reduced by 1 mg of membrane proteins per minute.

### 4.6. Statistical Analysis

All data presented were expressed as mean ± standard error (SE) or standard deviation (SD). The differences between means (*p*  ≤  0.05) were determined by one-way ANOVA followed by Duncan test post hoc using SigmaPlot 12 (Systat Software, Inc, Palo Alto, CA, USA).

## 5. Conclusions 

Our results suggest that the maintenance of the basal activity of NOXs in the leaves of the halophytic *E. salsugineum* plays a role in late acclimation responses to salt stress. The different expression patterns of the *RBOHD* and *RBOHF* genes under salinity in *E. salsugineum* and *A. thaliana* point to a modified regulation of these genes in the halophytic *E. salsugineum*, possibly through the ABA- and/or ethylene-dependent pathways.

## Figures and Tables

**Table 1:** Putative hormone *cis*-acting regulatory elements in *EsRBOHD*, *EsRBOHF*, *AtRBOHD*, and *AtRBOHF* promoters using the PLACE database.

Cis-Acting Regulatory Elements	Core Sequence	Hormone	Number of Elements			
			*EsRBOHD*	*EsRBOHF*	*AtRBOHD*	*AtRBOHF*
ERELEE4	AWTTCAAA	Ethylene	1 (+)	1 (−)	1 (+) 2 (−)	1 (−)
GT1CONSENSUS	GRWAAW	Salicylic acid	5 (+) 10 (−)	7 (+) 7 (−)	7 (+) 6 (−)	11 (+) 9 (−)
WBOXATNPR1	TTGAC	Salicylic acid	2 (+) 3 (−)	2 (−) 2 (−)	4 (+) 1 (−)	1 (+) 2 (−)
ASF1MOTIFCAMV	TGACG	Salicylic acid, Auxins	-	1 (−)	-	3 (−)
ARFAT	TGTCTC	Auxins	1 (+)	-	1 (−)	-
CATATGGMSAUR	CATATG	Auxins	1 (+) 1 (−)	-	-	-
CPBCSPOR	TATTAG	Cytokinins	1 (+)	1 (−)	2 (−)	1 (+) 3 (−)
WRKY71OS	TGAC	Gibberellins	6 (+) 6 (−)	4 (+) 4 (−)	8 (+) 6 (−)	2 (+) 5 (−)
MYBGAHV	TAACAAA	Gibberellins	1 (−)	1 (+)	2 (−)	-
GAREAT	TAACAAR	Gibberellins	1 (−)	1 (+)	3 (−)	1 (−)
PYRIMIDINEBOXHVEPB1	TTTTTTCC	Gibberellins, ABA	-	1 (+)	-	1 (−)
DPBFCOREDCDC3	ACACNNG	ABA	1 (+)	2 (+)	2 (+) 2 (−)	1 (+)
RYREPEATVFLEB4	CATGCATG	ABA	-	1 (+) 1 (−)	-	-
MYB1AT	WAACCA	ABA	1 (+) 1 (−)	1 (+)	1 (+) 2 (−)	1 (+)
MYCATRD22	CACATG	ABA	1 (+)	1 (+)	1 (+)	-
LTRECOREATCOR15	CCGAC	ABA	-	-	1 (+)	1 (+) 1 (−)

A + sign within brackets indicates the location of a motif on the presented promoter sequence; a - sign within brackets denotes the position of a motif on the complementary strand of the presented promoter sequence; N = A/C/G/T; R = G/A; W= A/T.

## Data Availability

All relevant data of this article are available within the manuscript and its Supplementary Materials.

## Supplementary Materials

The following are available online at https://www.mdpi.com/article/10.3390/ijms221910341/s1, Figure S1: The multiple alignment of RBOHD and RBOHF amino acids in *Arabidopsis thaliana* and *Eutrema salsugineum* constructed using the EMBL-EBI Clustal OMEGA tool (www.ebi.ac.uk/Tools/msa/clustalo/; accessed on 20 March 2020). Table S1: Selected parameters of tap water used for irrigation according to the information provided by the Kraków waterworks. Table S2: PCR primers used for quantitative RT-PCR.
