# Supplementary material for: Differential Regulation of NAPDH Oxidases in Salt-Tolerant Eutrema salsugineum and Salt-Sensitive Arabidopsis thaliana
Source: Int J Mol Sci. 2021 Sep 25;22(19):10341. doi: 10.3390/ijms221910341 (PMC8508936; doi:10.3390/ijms221910341)
Supplement: Supplementary file 1 [file ijms-22-10341-s001.zip › ijms-1379613-supplementary.pdf]

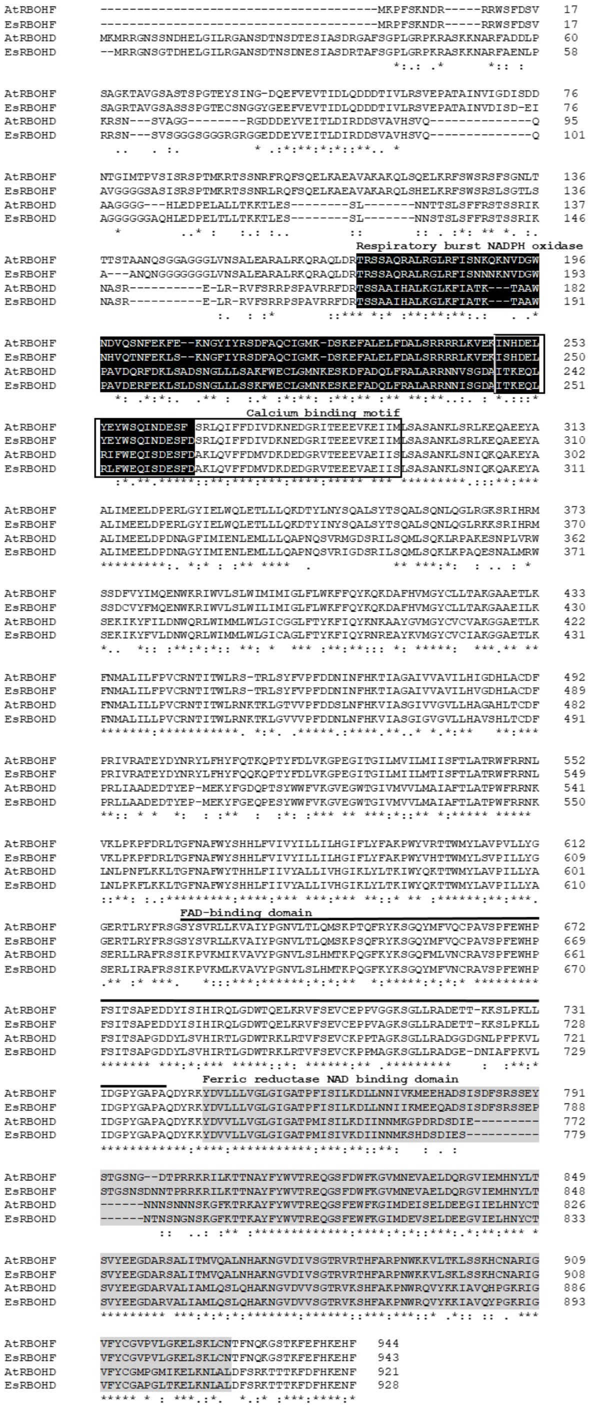

Fig. S1. The multiple alignment of RBOHD and RBOHF amino acids in *Arabidopsis thaliana* and *Eutrema salsugineum* constructed using EMBL-EBI Clustal OMEGA tool (https://www.ebi.ac.uk/Tools/msa/clustalo/). (*) indicate full conservation among all sequences, (:) highly conservative exchanges and (.) conservative exchanges of amino acids. The black color highlights the Respiratory burst NADPH oxidase domain (PF08414). The FAD-binding domain (PF08022) is indicated with a straight line above the alignment. The grey color marks Ferric reductase NAD binding domain (PF08030) and the box identifies calcium binding motif (cd00051). The conserved motifs were identified using NCBI Batch CD-Search online tool (https://www.ncbi.nlm.nih.gov/Structure/bwrpsb/bwrpsb.cgi).

Table S1. Selected parameters of tap water used for irrigation according to the information provided by the Kraków waterworks (Wodociągi Miasta Krakowa S.A.) at website https://en.wodociagi.krakow.pl/.

pH	7.6
NH_4^+	0.017 mg/dm^3
NO_2^-	< 0,01 mg/dm^3
NO_3^-	15,6 mg/dm^3
Cl^-	39,7 mg/dm^3
Ca	96 mg/dm^3
Mg	12 mg/dm^3
Fe	< 0,025 mg/dm^3
Cu	< 0,003 mg/dm^3
Cr	< 0,002 mg/dm^3
Cd	< 0,00045 mg/dm^3
Chloroform	< 0,3 μg/dm^3

Table S2. PCR primers used for quantitative RT-PCR.

Gene name	Gene reference	5´ – 3´ primer sequence	Product size (bp)	Amplific. Efficiency (%)
AtRBOHD	At5g47910	GGTTAAGATGATCAAGGTGGCT<br>TCCTTGTGGCTTCGTCATGT	70	100
AtRBOHF	At1g64060	ACGGGGTGTGATAGAGATGC<br>GCAAAGTGTGTTCTGACCCT	150	104
AtAT5G	At5g13440	CTTTCCCGTGACCATACCCT<br>AGCAAAACCTCTTATAACGCTCC	120	100
EsRBOHD	Thhalv10003619m	CACTTCGCCAAACCCAACTG<br>GAAGACTCCGATTCGTTTGCC	78	99
EsRBOHF	Thhalv10023240m	ATGGAAGAGCAAGCGGATTC<br>GCCTTGTTCTCTTGTGACCC	147	100
EsAPT1	Thhalv10008627m	GTCCGACTACTTGAGCGAGT<br>CCCTTCCCTTACACTCTGGT	79	100
